# Loop replacements with gut-binding peptides in Cry1Ab domain II enhanced toxicity against the brown planthopper, *Nilaparvata lugens* (Stål)

**DOI:** 10.1038/srep20106

**Published:** 2016-02-01

**Authors:** Ensi Shao, Li Lin, Chen Chen, Hanze Chen, Haohan Zhuang, Songqing Wu, Li Sha, Xiong Guan, Zhipeng Huang

**Affiliations:** 1 Key Laboratory of Biopesticide and Chemical Biology, Ministry of Education, Fujian Agriculture and Forestry University, 350002 Fuzhou, Fujian, PR China; 2 China National Engineering Research Center of JUNCAO Technology, Fujian Agriculture and Forestry University, 350002 Fuzhou, Fujian, PR China

## Abstract

*Bacillus thuringiensis* (Bt) Cry toxins have been used widely in pest managements. However, Cry toxins are not effective against sap-sucking insects (Hemiptera), which limits the application of Bt for pest management. In order to extend the insecticidal spectrum of Bt toxins to the rice brown planthopper (BPH), *Nilaparvata lugens*, we modified Cry1Ab putative receptor binding domains with selected BPH gut-binding peptides (GBPs). Three surface exposed loops in the domain II of Cry1Ab were replaced with two GBPs (P2S and P1Z) respectively. Bioassay results showed that toxicity of modified toxin L2-P2S increased significantly (~9 folds) against BPH nymphs. In addition, damage of midgut cells was observed from the nymphs fed with L2-P2S. Our results indicate that modifying Cry toxins based on the toxin-gut interactions can broaden the insecticidal spectrum of Bt toxin. This method provides another approach for the development of transgenic crops with novel insecticidal activity against hemipteran insects and insect populations resistant to current Bt transgenic crops.

Transgenic plants carrying Cry toxins of *Bacillus thuringiensis* (Bt) are widely used to control major lepidopteran and coleopteran insect pests^1^,^2^. However, Cry toxins are only effective against lepidopteran, coleopteran, and dipteran insect pests, as well as nematodes^3^,^4^. Cry toxins with significant insecticidal activity against hemipteran insects were hardly identified^5^,^6^ although one crystal protein (TIC807) was reported to impact the development and survival of *Lygus hesperus* nymphs^7^. Hence, modification of insecticidal toxins, such as Bt toxins, for the control of hemipteran pests has great promise to meet the challenge of pest management in the future.

The engineering of Cry toxins based on the understanding of the mode of action is an approach to broaden their insecticidal spectrum. The mode of action of Cry toxins is complex^4^,^8^,^9^. The current understanding of the action mode of Cry toxins indicates that after Bt inclusions are solubilized in the digestive tract of target insects, the Cry protoxins are then activated and bind subsequently to the receptors for the toxins on the epithelium of the insect midgut before the activated toxins insert into cell membranes and lyse the cells^8^. Known Cry toxin receptors include aminopeptidase N (APN), alkaline phosphatase (ALP), cadherin-like proteins and ATP-binding cassette (ABC) transporters^4^,^8^,^10^. Furthermore, functional domains that determine potential interactions between toxins and host gut cells in Cry action mode have been predicted and tested experimentally in several cases^11^,^12^, and these provide a basis for Cry toxin engineering to improve Cry-host interactions.

Modification of Cry functional domains has been reported to improve toxicity^13^,^14^,^15^,^16^. Mehlo *et al.*, (2005) constructed transgenic plants expressing ricin B-chain combined Cry1Ac toxin. The transgenic plants were resistant to a wide range of insects^16^. Ishikawa *et al.*, (2007) used T7 phages to construct a library of loop 2 in the Cry1Aa toxin domain II to screen a loop 2 mutated Cry1Aa toxin, which resulted in a 6-fold increase in toxicity against *Bombyx mori* larvae^17^. Lassner and Bedbrook used DNA shuffling to combine the segments of Cry1Ca and Cry1Ab toxins and discovered a novel Bt variant that showed 3.8-fold improved toxicity against *Spodoptera exigua*^18^. Furthermore, after a triple site-directed mutagenesis in the domain II loop 2, a modified Cry3A toxin showed 10- and 2-fold higher toxicity against *Tenebrio molitor* and *Leptinotarsa decemlineata*, respectively^19^.

Previous investigations of activation and stability of Cry1A toxins in the intestinal environment of *Acyrthosiphon pisum*, a hemipteran insect, showed that Cry1Ac protoxin is activated by gut proteases^5^. The activated Cry1Ac generally showed a high toxicity against *Ostrinia nubilalis* larvae, but extremely low toxicity against *A. pisum* nymphs^5^. A similar study on the proteolytic processing of Cry1Ab by gut proteases of rice brwon planthopper (BPH), *Nilaparvata lugens* also showed that a fully activated Cry1Ab exhibited 100% insecticidal activity against larvae of diamondback moth (DBM), *Plutella xylostella* (Linnaeus), but had a significantly lower toxicity to BPH nymphs^6^. In both studies, lower binding affinities of the activated Cry toxin to brush border membrane vesicles (BBMV) were observed, supporting the hypothesis that some Cry toxins are activated in the gut of hemipteran insects, but that the activated toxins could not interact with potential receptors. Indeed, it has been shown that APN, ALP and cadherin-like proteins of aphids have only limited similarities to their orthologs in other insect species^20^. Likewise, we observed that potential Cry receptors of BPH have low sequence similarity to their orthologs in insects that are susceptible to Cry toxins (Shao *et al.*, unpublished result).

A toxin modification approach has recently been conducted by modifying Bt Cyt2Aa toxins through either adding or replacing amino acids in one of the seven loops with a pea aphid gut binding peptide (GBP3.1)^21^,^22^. The resultant modified Cyt2Aa showed enhanced binding activity to the gut and increased toxicity to both the pea aphid and the green peach aphid, *Myzus persicae*. The most significant enhancement in toxicity was observed by inserting GBP3.1^22^ into the loop 3 of Cyt2Aa, which resulted in more than a 50-fold higher toxicity against *A. pisum* nymphs as compared to native Cyt2Aa^21^.

BPH is one of the most notorious rice insect pests in eastern and southeastern Asia^23^, which feeds mainly on the stem and assimilates from the phloem of rice^24^, leading to wilted tillers and withered leave^25^. In addition, BPHs are key vectors for transmitting rice grassy stunt virus and ragged stunt virus, which can cause a severe decline in rice production^26^. A number of genetically engineered insect-resistant rice varieties expressing Bt toxins have been developed, which are effective primarily at managing lepidopteran pests such as *Chilo suppressalis*, *Tryporyza incertulas* and *Cnaphalocrocis edinalis*^2^,^27^,^28^. However, Bt transgenic rice has little impact on BPH^29^. In fact, the suppression of major lepidopteran and coleopteran pests may lead to outbreaks of BPH^30^. Potential outbreaks of BPH and other hemipteran pests would compromise benefits of Bt transgenic technology for pest management^31^,^32^.

We have shown previously that Cry1Ab could be *in vitro* proteolytically processed by the gut proteases of BPH and retained 100% activity against its target insect DBM^6^. Here we replaced Cry1Ab domian II loop regions with short peptides that could bind to the BPH gut^33^,^34^. Resulted toxins exhibited increased toxicity against BPH nymphs. Our work demonstrates that substituting Cry1Ab functional domains with GBPs could significantly increase toxicity of the Bt toxin against BPH.

## Results

### Binding of P1Z and P2S to BPH BBMV

**Figure 1:**
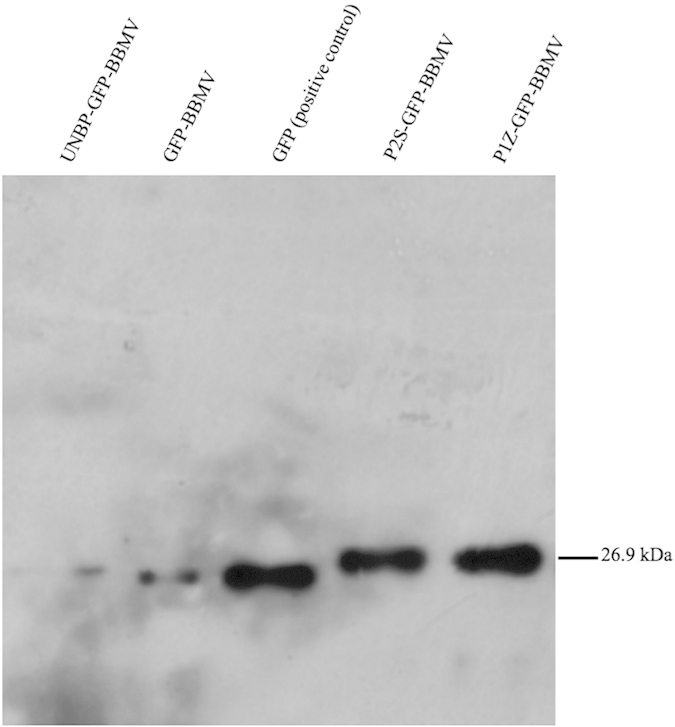
*In vitro* binding assay of P1Z and P2S with BPH BBMV. Showing binding activity of P1Z-GFP and P2S-GFP to the BPH BBMV. Only P1Z-GFP and P2S-GFP bound strongly to the BPH BBMV, while only a very faint signal was observed from either UNBP-GFP-BBMV or GFP-BBMV (negative control).

P1Z and P2S are two BPH gut-binding peptides, screened and selected from phage display library either by *in vitro* or *in vivo* method^33^,^34^. Both P1Z and P2S contain 9 amino acids (P1Z: CHLPRLPQC; P2S: CLMSSQAAC). The two peptides and a control peptide, known not to bind to the BPH gut (UNBP: CIQPNLNHC), were fused with GFP and expressed as P1Z-GFP, P2S-GFP and UNBP-GFP fusion proteins^33^,^34^. Protein binding assays confirmed the binding of the two BPH gut peptides to BPH gut membrane (Fig. 1). An isolated product with a M_r_ of ~27 kDa was observed in the P1Z-GFP-BBMV and P2S-GFP-BBMV samples, which is about the size of GFP control (positive control). In contrast, very weak signals were seen in the samples of UNBP-GFP-BBMV and GFP-BBMV (negative control). These results showed that the two peptides (P1Z and P2S) selected through biopanning of phage peptide library could bind to the BPH gut membrane, and therefore are good candidates for modifying of Cry1Ab.

### Stability of the modified Cry toxins after exposure to BPH gut proteases

The replacement of 3 loops (278RG279, 335 RRPFNIGINNQ 345, 401 SMFRSGFSNSSVS 413) located in domain II on the surface of Cry1Ab toxin resulted in six modified Cry1Ab proteins (e.g. L1-P1Z, L1-P2S for loop 1, L2-P1Z, L2-P2S for loop 2 and L3-P1Z, L3-P2S for loop 3 substitutions). The six modified Cry toxins were tagged with GST and recombinant expression yielded expected ~133 kDa recombinant proteins after the removal of GST taq, confirming that the loop modified Cry1Ab proteins were successfully expressed.

**Figure 2:**
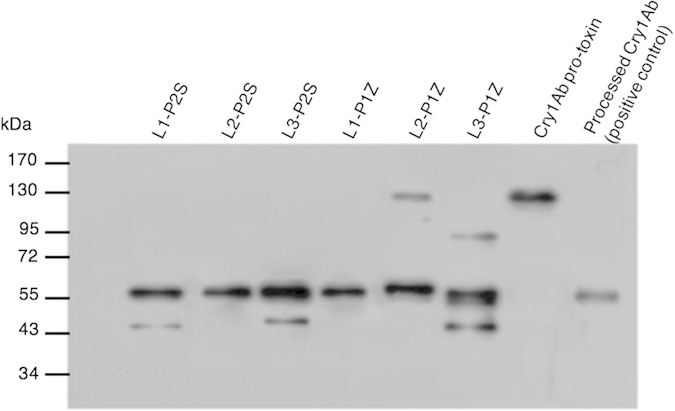
*In vitro* proteolytic processing of modified Cry proteins by BPH gut proteases. Processed Cry1Ab fragments were detected by western blot with a rabbit polyclonal Cry1Ab serum as the primary antibody and anti-rabbit-IgG conjugated to HRP as the secondary antibody. Cry1Ab protoxins processed by trypsin and without processing were used as positive and negative controls, respectively.

The results of *in vitro* proteolytic processing of each modified Cry toxin by BPH gut proteases showed that a majority of the modified protoxins were converted from about 133 kDa to 60 kDa as expected (Fig. 2). However, small proportions of the loop 3 substituted Cry1Ab (L3-P2S and L3-P1Z) and loop 1 substituted Cry1Ab (L1-P2S) were further processed into approximately 45 kDa fragments, which might be due to the exposure of additional protease cleavage site of the modified loop 3 or loop 1 in the toxin structure after the loop replacement. In addition, the activation of L2-P1Z seemed to be slower than the others as indicated by the presence of a ~133 protoxin and ~90 kDa fragment after a 16 h incubation period with the BPH proteases, supporting the conclusion that the modification reduced the efficiency of proteolytic processing.

### Bioassay of the modified Cry toxins against BPH and DBM

To test whether the modification could affect insecticidal activity of toxins, the toxicity of the modified Cry1Ab toxins against Cry1Ab target lepidopteran insects were firstly tested. We expected decrease in toxicity of the modified Cry1Ab toxins against the lepidopteran insect since the replacement of receptor binding region may obstruct the binding between Cry1Ab toxins and receptors in target insect gut. The data showed that all loop-modified Cry1Ab toxins showed statistically decrease in toxicity against DBM larvae. The most significant decrease in toxicity, which corresponds to an increase of LC_50_, was observed from the replacement of loop 2, with LC_50_ = 30.48 μg/mL for L2-P2S and LC_50_ = 33.39 μg/mL for L2-P1Z, an approximately 37-fold higher than the LC_50_ (0.88 μg/mL) of native Cry1Ab (Table 1).

The insecticidal activity of the modified toxins against BPH nymphs was then tested. As expected, native Cry1Ab toxin showed very low toxicity against BPH nymphs (LC_50_ = 189.83 μg/mL). In contrast, all loop replaced Cry1Ab toxins showed certain degrees of activity. The LC_50_ of five modified Cry1Ab toxins, but L3-P1Z, were all statistically reduced, indicating an increase in toxicity. The strongest toxicity was observed from L2-P2S (replacement of loop 2 with P2S) with an LC_50_ of 21.54 μg/mL, which was nearly 9-fold less than that of Cry1Ab. Interestingly, all modified Cry proteins with P2S showed statistically higher activity against BPH than those with P1Z (only small overlap was found between 95% confident interval of L3-P2S and L2-P1Z) (Table 1). These results indicate that peptide P2S is more effective in triggering toxicity against BPH than engineering of Cry1Ab by peptide P1Z. Mortality of nymphs fed with high concentrations of BSA was around 13% (Table 2) and calculated as natural response rate in Probit analysis.

### Damage of BPH gut epithelial membrane by L2-P2S

**Figure 3:**
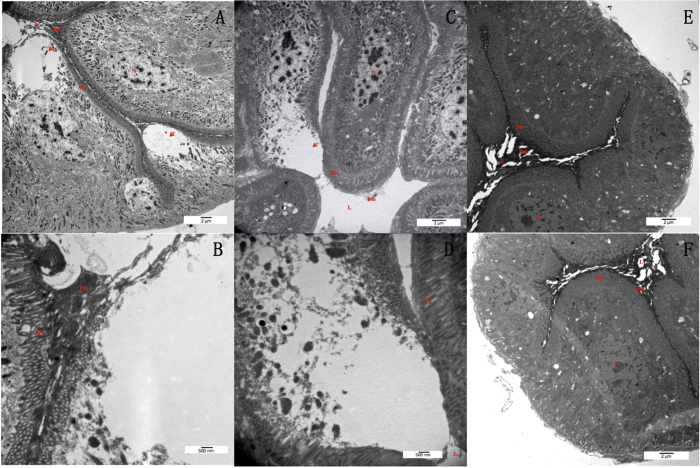
Observation of the damaged gut epithelial cells of BPH after ingestion of L2-P2S toxin. Gut samples from treatments in bioassay were prepared into ultrastructure samples for observation by transmission electron microscope. Panels (**A**–**D**) indicate ultrastructure of BPH gut epithelial cells ingested with L2-P2S (lysis cells were pointed by arrows). Panels (**E**,**F**) indicate ultrastructure of BPH gut epithelial cells ingested with Cry1Ab or PBS only respectively. Mv: microvilli; N: nucleus; Pm: peritropic matrix; L: lumen.

Bioassay showed that the domain II loop replacements of Cry1Ab significantly affected toxicity to BPH. Damage of BPH gut epithelial cell membrane after ingestion of L2-P2S, which showed highest insecticidal activity against BPH nymphs were detected. In the gut of BPH fed with L2-P2S, enlargement and lysis of some columnar cells were clearly observed along with the disintegration of the epithelial cells layer and the extrusion of cytoplasm into the lumen (Fig. 3A–D). In contrast, no damaged epithelial cells were observed in the samples prepared from BPHs fed with Cry1Ab or only PBS (Fig. 3E,F).

## Discussion

To manipulate the specificity of Cry toxins to new target insects requires changing the toxin to allow effective completion of the intoxication pathway in the gut, leading to disruption of the gut cells and eventually mortality of the target insects^8^,^13^. In 3-domain Bt Cry toxins, 3 loops, loops 1, 2 and 3, in the domain II on the surface of Cry toxins have been predicted to play significant roles in receptor binding^35^,^36^. In order to alter Cry toxins with improved insecticidal activity to BPH nymphs, in this study, we replaced amino acid residues of the 3 loops in domain II of Cry1Ab toxin with either the peptide P1Z or P2S. Results showed that 6 engineered Cry1Ab toxins with loop replacements in the domain II could be proteolytically processed into a ~60 kDa fragment by the gut proteases of BPH (Fig. 2), leading to higher mortality in BPH nymphs. Disruption of epithelium cells in the midgut tissue of BPH nymphs fed with L2-P2S was also observed by TEM (Fig. 3A–D). These results demonstrate that the loop swapping of the Cry1Ab binding domain with a GBP could significantly improve the insecticidal activity against new target insects. Our work on the modification of Cry1Ab may substantially improve the insecticidal efficacy for the control of hemipteran insect pests.

The insecticidal activity of the modified Cry1Ab was still lower compared to that in the lepidopteran target insect, the diamondback moth (Table 1). Although the activation of Cry1Ab protoxin by BPH gut protease was previously confirmed^6^, interaction of engineered toxins to potential receptors in BPH gut is still unclear. Understanding of the specific gut protein(s) that the modified Cry1Ab with a GBP binds to is required to further understand the interaction of the modified toxin with BPH.

Reduced stability of modified toxins when treated with BPH gut proteases was observed in this study (Fig. 2), which might partly explain the limited toxicity against BPH. We observed previously that Cry1Ab protoxin could be activated efficiently into ~60 kDa fragment by BPH gut proteases and the activated Cry toxin was stable when treated with BPH gut proteases^6^. However, modified toxins L1-P2S, L3-P2S and L1-P1Z have a further degradation of the immune-activated functional protein (Fig. 2). In the midgut of target lepidopteran insects, wild-type Cry1Ab protoxin is activated into a ~60 kDa protein mostly by trypsin, chymotrypsin and cysteine protease^37^. Although trypsin and cathepsins have been reported to exhibit a high transcriptome abundance in BPH gut^38^,^39^, more details of BPH gut proteases are needed to be studied to explain the reason of less stability of modified toxins.

Previous work on domain swapping for enhancement of insecticidal activity of the Cry toxins was achieved using the corresponding loops of different Cry toxins^40^,^41^. In this research, the loops 1, 2 and 3 of Cry1Ab domain II were replaced by non Cry toxin related gut binding peptide P2S or P1Z. The modified Cry1Ab with loop replacements showed enhanced insecticidal activity against BPH (Table 1). The replacement of loop 2 appeared to more effectively improve the toxicity of Cry1Ab against BPH. However, the modified toxin reduced toxicity to DBM for up to 37 folds (Table 1). The structural model of Cry1A toxin indicates that loop 2 is the most exposed region in domain II^42^, which may explain why the toxicity of loop 2 replaced Cry1Ab was affected most significantly. On the other hand, loop 1 of Cry1Ab has only 2 amino acids (^310^RG^311^). Its potential function in receptor binding requires to be understood. Replacement of loop 1 with a BPH GBP increased toxicity of Cry1Ab against BPH nymphs up to 5 folds, and in contrast reduced toxicity against DBM larvae more than 6 folds. Swapping of loop α-8 of domain II and β-16 of domain II with GBPs significantly affected toxin stability (results not shown), demonstrating the importance of these two loops in maintaining the stability of Cry1Ab.

Chouglue *et al.*, (2013) observed that modification of Cyt2Aa toxin by addition of peptides exhibited higher toxicity against pea aphids, compared to replacement of the loops with the peptides. In order to maintain structure of Cry1Ab toxin and stability of the modified toxin structure, we conducted the toxin modification with loop replacement, instead of additional peptide insertion into the loops. Future study will test whether insertion of additional peptides affects stability of the toxin and alters the toxin toxicity.

Binding of activated toxins with proper receptors located in the midgut of target insect has been reported as an essential step in the mechanism of Cry toxins. In this study, although damaged gut epithelium cells of BPH nymphs fed with L2-P2S toxin was observed, the gut proteins interacting with L2-P2S toxin are remained to be identified. The TEM observations in this study (Fig. 3A–D) did not show extensive damage of epithelial cells in the gut of BPH. Major damage to the gut was observed not only at the apical tip but also at the base of microvilli, showing extrusion of cytoplasm. Therefore, if the modified toxins bind to putative receptors, the distribution of the receptor molecules may not be abundant in the midgut. This may explain why the modified Cry1Ab had only limited insecticidal activity to BPH. It is also possible that the modified toxins bound to molecules that are not conventional Cry toxin receptors and such molecules may have low expression abundance or only exist at specific location in BPH gut. The mechanism of the insecticidal activity in this case may be different from the well-known action mode of Cry toxins. Future studies are to identify potential Cry receptors in BPH and to modify Cry toxins based on toxin-receptor interactions, which will be critical for the development of effective Cry toxins against BPH.

In conclusion, replacement the loops in Cry1Ab with a GBP in this study altered insecticidal activity of Cry1Ab toxin to non-target insect BPH with increase insecticidal efficacy. Although the mechanism of engineered toxins in BPH gut is still unclear, this study demonstrated an approach to alter the insecticidal spectrum of Bt toxins. Further research is required to identify the binding proteins of L2-P2S in the gut of BPH. Insect populations resistant to Bt toxin have been reported in a number of insect pests, such as cabbage looper (*Trichoplusia ni*)^43^, western corn rootworm (*Diabrotica virgifera virgifera*)^44^, beet armyworm (*Spodoptera exigua*)^45^ and fall armyworm (*Spodoptera frugiperda*)^46^. Reduced toxin-receptor binding affinity due to mutations of the receptor molecules was one of the most important reasons for the development of resistance^47^,^48^. Use of receptor binding peptides (RBP) to replace the toxin-binding domain may be a solution for overcoming Cry toxin resistance.

## Materials and Methods

### Insects

BPHs used for this research were provided by the Institute of Plant Virology, Fujian Agriculture and Forestry University, Fuzhou, Fujian, China. The planthoppers were maintained on rice (*Oryza sativa L.*) seedlings, and kept in a growth chamber at 28 °C and 14 h : 10 h (light : dark). A laboratory strain of DBM^49^, which is sensitive to Cry1Ab, was raised on radish sprouts (*Raphanus sativus*) under the same conditions as those for BPH.

### Preparation of BPH gut BBMV

BPH guts were isolated from 3–4 instars nymphs to prepare gut BBMV using the methods described previously^5^,^50^. Briefly, 1500 of BPH nymphs gut tissues were collected in 1.5 mL MET buffer A (0.3 M Mannitol, 5 mM EGTA, 17 mM Tris-HCl pH7.5) with a protease inhibitor, PI [Roche, US] cocktail (one tablet in 50 mL buffer A) to prevent protein degradation, stored at −80 °C until use. The isolated guts were homogenized in a hand homogenizer in ice cold MET buffer A. From the homogenate, BBMV were prepared by differential precipitation using MgCl_2_. Extracted BBMV pellets were resuspended in ice cold MET buffer A with protease inhibitors, aliquoted, and then frozen with liquid nitrogen and stored at −80 °C until use. The concentration of the BBMV extractions was determined by the Bradford method using a Coomassie (Bradford) Protein Assay kit (Biomiga, China). Alkaline phosphatase (ALP) enzyme activity was used to examine the BBMV quality. The testing for ALP activity was determined using PNPP (P-nitrophenyl Phosphate, Sigma) as a substrate by measuring OD_595_ in a microplate reader (Bio-Red, iMark). The rate of OD_595_ was then converted to ALP activity (U/mg/mL).

### Binding assay of GBPs to BPH BBMV

Binding of peptide-GFP fusions^33^,^34^ to BPH gut BBMV was conducted by qualitative binding assay as described previously^51^. In brief, 20 μg of BBMV was mixed with 50 μL peptide-GFP fusion protein (100 μg/mL) and 50 μL binding buffer (PBS, 0.1% BSA, 0.1% Tween 20, pH 7.6). The mixtures were incubated at 28 °C for 1 h and then centrifuged for 10 min at 11,000 ×g at 4 °C. The supernatants were discarded to remove unbound proteins and the pellets were washed twice with 100 μL binding buffer and centrifuged for 10 min (11,000 ×g at 4 °C). The protein pellets were resuspended in 10 μL PBS (pH 7.6) and 2 μL 5× Laemmli buffer^52^, and heated immediately in 98 °C water for 5 min to denature the proteins. Protein samples were loaded onto a 12% SDS-PAGE gel and separated by electrophoresis. Then, proteins were transferred onto PVDF membranes for immunostaining.

To detect the GFP tag in the peptide-GFP fusion proteins, monoclonal mouse anti-GFP antibody (Sigma, US 1:2000 dilution) and horse radish peroxidase (HRP)-conjugated rabbit anti-mouse IgG (Sigma, US 1:80000 dilution) were used as the primary and the secondary antibodies, respectively. GFP-tagged peptides and BPH BBMV incubated with GFP were used as positive and negative controls, respectively. The blots were overlapped with BeyoECL plus Chemiluminescent HRP detection reagent (Beyotime, China) for 1 min, followed by detection of luminescence on X-ray film using standard procedures to visualize signals.

### Replacement of domain II loops of Cry1Ab

To construct Cry1Ab mutants with a GBP sequence substitution, a 1448 bp *Spe* I *–Mun* I fragment located between 177–1624 bp of the Cry1Ab gene and containing the 3 loops of the domain II sequence was amplified by PCR and cloned into a pMD-18T vector (TaKaRa, China), resulting in a pMF-18T plasmid.

Loop sequence substitutions were obtained by overlapping and extension PCR^21^ using pMF-18T as templates. Primers used for the generation of loop replacements in PCR fragments are listed in Table 3. Briefly, peptide sequences for loop replacements were encoded in primers and used for the generation of mutated *Spe* I *–Mun* I fragments through overlapping PCR. The mutated *Spe* I *–Mun* I PCR fragments were digested by *Spe* I and *Mun* I restriction enzymes, and then ligated into pGST-Cry1Ab plasmids, which were linearized with corresponding enzymes by standard molecular methods. Sequences of the obtained loop substituted clones (pGST-L1-P2S, pGST-L2-P2S, pGST-L3-P2S, pGST-L1-P1Z, pGST-L2-P1Z and pGST-L3-P1Z) were verified by DNA sequencing (TaKaRa, China). The mutated plasmids were then transformed into *Escherichia coli* BL21 (DE3) cells for Cry protein expression.

### Proteolytic processing of Cry proteins

Samples of 3^rd^- and 4^th^-instar BPH nymphs were dissected in distilled water to isolate the guts. The detached guts and contents were placed in 100 μL PBS (pH 7.0) and stored at −20 °C. Gut proteases were prepared as described previously^5^. In brief, ~500 gut fragments were homogenized in 500 μL PBS (pH 7.0) and the homogenates were centrifuged at 25,000 ×g for 20 min at 4 °C. The pellets containing proteases associated with the gut membranes were resuspended in 500 μL PBS (pH 7.0). The protein concentration of each sample was measured using the Bradford method.

To test the proteolytic processing of Cry and mutated protoxins by BPH gut proteases, 250 μL membrane-bound protease preparation was mixed with 3 mM of EDTA and 3 mM of cysteine and incubated at room temperature for 1 h to activate cysteine protease activity^5^. Thirty microliters of the activated cysteine proteases were mixed with the protoxins in a ratio of 5:1 (protease solution : protoxin, w/w). The protease-Cry mixtures were incubated at 28 °C for 16 h with moderate shaking (~40 rpm). To terminate the reaction, 10 μL 5× Laemmli buffer was added to each of the mixtures after 16 h. Results of processed Cry peptides were determined by immunoblot analysis as described previously.

### Bioassay of Cry toxins

The toxicity to 3^rd^-instar BPH nymphs was tested by membrane feeding with various concentrations (10, 25, 50, 100 and 200 μg/mL) of Cry toxins and BSA (control, 100 μg/mL, 200 μg/mL and 300 μg/mL) in 100 μL of complete artificial liquid diet D-97^53^. The bioassays were set up in duplicates with 10 nymphs per replicate and repeated six times. The bioassays were performed in a growth chamber at 28 °C and 14 h : 10 h (light : dark) for 3 days with the toxin-diet replaced every 24 h. Mortality of each treatment was recorded after 72 h.

The mutated Cry toxins also were tested for their toxicity in lepidopteran insects. Methods used for the bioassay of lepidopteran larvae of DBM were modified from a leaf residue bioassay^54^. The 3^rd^-instar larvae were fed with protoxins (0.24, 1.2, 2.4, 10, 20 and 55 μg/mL, respectively) of Cry toxins on leaf dishes (13 cm^2^). Larvae fed on buffer only (PBS with 0.2% Triton-100×) were used as a negative control. Environmental conditions for the bioassay were the same as those used in the BPH bioassay. Mortality was recorded after 48 h. Treatments were duplicated and repeated 6 times. Probit analysis of mortality data to estimate the LC_50_ and 95% confidence limits (CL) was carried out by the online data analysis tool (http://www.xuru.org/st/DS.asp) and SPSS software (version 22.0.0).

### Sample preparation for TEM

The 3^rd^ instars of BPH nymphs were either fed on a single concentration (20 μg/mL) of Cry1Ab in feeding buffer (PBS containing 25% sucrose, pH 7.0) or fed on D-97 artificial diet only (control) by membrane feeding. Digestion tissue of BPH nymphs was constructed by esophagus, fore-diverticulum, midgut, hindgut and malpighian tube^39^,^55^. Because gut tissue of BPH nymphs began to disintegrate in 48 h and weak gut tissue was difficult to prepare TEM samples. Ten BPH nymphs from all replicates were dissected to isolate their digestion tissues in 24 h, following with the removal of esophagus, fore-diverticulum, hindgut and malpighian tube. Isolated midguts were rinsing at PBS for three times. The midgut tissues were fixed immediately in 2% paraformaldehyde, 2.5% glutaraldehyde, 0.05 M cacodylate buffer, pH 7.1. Fixed tissues were embedded in Epon 812 (Yue, 1968). Sections (50–90 nm) were sliced using glass knives (silver to gold interference colors), stained with uranyl acetate for 1 h and followed by treatment with lead oxide for 3 min. Transversal sectioned gut samples were observed by transmission electron microscopy (TEM) (HITACHI H-7650 Electron Microscope).

## Additional Information

**How to cite this article**: Shao, E. *et al.* Loop replacements with gut-binding peptides in Cry1Ab domain II enhanced toxicity against the brown planthopper, *Nilaparvata lugens* (Stål). *Sci. Rep.* **6**, 20106; doi: 10.1038/srep20106 (2016).

## Figures and Tables

**Table 1:** Susceptibility of BPH and DBM to modified Cry protein.

Cry toxins	BPH			DBM		
	LC_50_ (95% CI, μg/mL)	Slope	Χ^2^ (*df*, P)	LC_50_ (95% CI, μg/mL)	Slope	Χ^2^ (*df*, P)
Cry1Ab	189.83 (156.58–247.74)	1.51 (±0.38)	1.263 (3, 0.738)	0.88 (0.74–1.02)	2.00 (±0.40)	6.934 (4, 0.139)
L1-P2S	37.82 (30.20–46.38)	1.01 (±0.24)	2.117 (3, 0.549)	5.89 (3.98–8.37)	1.82 (±0.43)	7.091 (4, 0.131)
L2-P2S	21.54 (17.83–25.31)	1.59 (±0.27)	2.926 (3, 0.403)	30.48 (23.98–40.92)	1.47 (±0.32)	5.023 (4, 0.285)
L3-P2S	37.47 (19.82–61.20)	1.10 (±0.26)	7.181 (3, 0.066)	13.72 (11.16–16.75)	1.80 (±0.39)	3.887 (4, 0.422)
L1-P1Z	137.77 (116.63–169.43)	1.52 (±0.39)	3.995 (3, 0.262)	6.84 (5.63–8.21)	2.19 (±0.31)	4.019 (4, 0.403)
L2-P1Z	77.45 (56.20–112.59)	1.61 (±0.35)	5.908 (3, 0.116)	33.39 (27.32–42.97)	1.63 (±0.24)	6.076 (4, 0.194)
L3-P1Z	231.72 (182.03–330.95)	1.36 (±0.57)	4.506 (3, 0.212)	18.05 (14.68–22.33)	1.94 (±0.46)	4.905 (4, 0.297)

CI, confidence interval.

LC_50_, 50% lethal concentration the LC_50_ value and their 95% fiducial limits were assessed by Probit analysis using SPSS (version 22.0.0) and the online statistical tools (http://www.xuru.org/st/DS.asp).

**Table 2:** Mortatlity of BPHS nymphs fed with high concentration of BSA.

Concentration of BSA (μg/mL)	Mortality (±SE)
100	12% (±3%)
200	13% (±4%)
300	13% (±2%)

**Table 3:** Primers to replace three loops in domain II of Cry1Ab by P1Z or P2S respectively.

Primer	Nucleotide sequence
MF-F	5′-ACTAGTTGATATAATATGGGGAATTT-3′
MF-R	5′-CAATTGATGTATGGAATTGTAAA-3′
P2Sloop1-R	5′-gcacgccgcctgcgacgacatcaaacaATGAGCATCCGTATAGATGGT-3′
P2Sloop1-F	5′-tgtttgatgtcgtcgcaggcggcgtgcGAATATTATTGGTCAGGGCA-3′
P2Sloop2-R	5′-gcacgccgcctgcgacgacatcaaacaATATAAAGTGGACGATAATGTTCTA-3′
P2Sloop2-F	5′-tgtttgatgtcgtcgcaggcggcgtgcCAACTATCTGTTCTTGACGGG-3′
P2Sloop3-R	5′-gcacgccgcctgcgacgacatcaaacaAACATGGCTTAATCGATGACTA-3′
P2Sloop3-F	5′-tgtttgatgtcgtcgcaggcggcgtgcATAATAAGAGCTCCTATGTTCTCTT-3′
P1Zloop1-R	5′-gcactgtggaagtcggggaaggtgacaATGAGCATCCGTATAGATGGT-3′
P1Zloop1-F	5′-tgtcaccttccccgacttccacagtgcGAATATTATTGGTCAGGGCA-3′
P1Zloop2-R	5′-gcactgtggaagtcggggaaggtgacaATATAAAGTGGACGATAATGTTCTA-3′
P1Zloop2-F	5′-tgtcaccttccccgacttccacagtgcCAACTATCTGTTCTTGACGGG-3′
P1Zloop3-R	5′-gcactgtggaagtcggggaaggtgacaAACATGGCTTAATCGATGACTA-3′
P1Zloop3-F	5′-tgtcaccttccccgacttccacagtgcATAATAAGAGCTCCTATGTTCTCTT-3′

Enzyme site *Spe* I and *Mun* I is underlined and lower case text indicating nucleotide sequence of peptide P1Z and P2S.
